# Neuropeptide Y receptor activation preserves inner retinal integrity through PI3K/Akt signaling in a glaucoma mouse model

**DOI:** 10.1093/pnasnexus/pgae299

**Published:** 2024-07-26

**Authors:** Viswanthram Palanivel, Vivek Gupta, Nitin Chitranshi, Ole Tietz, Roshana Vander Wall, Reuben Blades, Kanishka Pushpitha Maha Thananthirige, Akanksha Salkar, Chao Shen, Mehdi Mirzaei, Veer Gupta, Stuart L Graham, Devaraj Basavarajappa

**Affiliations:** Faculty of Medicine, Health and Human Sciences, Macquarie Medical School, Macquarie University, North Ryde, Sydney, NSW 2109, Australia; Faculty of Medicine, Health and Human Sciences, Macquarie Medical School, Macquarie University, North Ryde, Sydney, NSW 2109, Australia; Faculty of Medicine, Health and Human Sciences, Macquarie Medical School, Macquarie University, North Ryde, Sydney, NSW 2109, Australia; Faculty of Medicine, Health and Human Sciences, Dementia Research Centre, Macquarie Medical School, Macquarie University, North Ryde, Sydney, NSW 2109, Australia; Faculty of Medicine, Health and Human Sciences, Macquarie Medical School, Macquarie University, North Ryde, Sydney, NSW 2109, Australia; Faculty of Medicine, Health and Human Sciences, Dementia Research Centre, Macquarie Medical School, Macquarie University, North Ryde, Sydney, NSW 2109, Australia; Faculty of Medicine, Health and Human Sciences, Dementia Research Centre, Macquarie Medical School, Macquarie University, North Ryde, Sydney, NSW 2109, Australia; Faculty of Medicine, Health and Human Sciences, Macquarie Medical School, Macquarie University, North Ryde, Sydney, NSW 2109, Australia; Microscopy Unit, Faculty of Science and Engineering, Macquarie University, North Ryde, Sydney, NSW 2109, Australia; Faculty of Medicine, Health and Human Sciences, Macquarie Medical School, Macquarie University, North Ryde, Sydney, NSW 2109, Australia; School of Medicine, Deakin University, Geelong, VIC 3216, Australia; Faculty of Medicine, Health and Human Sciences, Macquarie Medical School, Macquarie University, North Ryde, Sydney, NSW 2109, Australia; Save Sight Institute, The University of Sydney, Sydney, NSW 2000, Australia; Faculty of Medicine, Health and Human Sciences, Macquarie Medical School, Macquarie University, North Ryde, Sydney, NSW 2109, Australia

**Keywords:** neuropeptide Y, glaucoma, intraocular pressure, neuroprotection, PI3K/Akt signaling

## Abstract

Neuropeptide Y (NPY), an endogenous peptide composed of 36 amino acids, has been investigated as a potential therapeutic agent for neurodegenerative diseases due to its neuroprotective attributes. This study investigated the neuroprotective effects of NPY in a mouse model of glaucoma characterized by elevated intraocular pressure (IOP) and progressive retinal ganglion cell degeneration. Elevated IOP in mice was induced through intracameral microbead injections, accompanied by intravitreal administration of NPY peptide. The results demonstrated that NPY treatment preserved both the structural and functional integrity of the inner retina and mitigated axonal damage and degenerative changes in the optic nerve under high IOP conditions. Further, NPY treatment effectively reduced inflammatory glial cell activation, as evidenced by decreased expression of glial fibrillary acidic protein and Iba-1. Notably, endogenous NPY expression and its receptors (NPY-Y1R and NPY-Y4R) levels were negatively affected in the retina under elevated IOP conditions. NPY treatment restored these changes to a significant extent. Molecular analysis revealed that NPY mediates its protective effects through the mitogen-activated protein kinase (MAPK) and PI3K/Akt signaling pathways. These findings highlight the therapeutic potential of NPY in glaucoma treatment, underscoring its capacity to preserve retinal health, modulate receptor expression under stress, reduce neuroinflammation, and impart protection against axonal impairment.

**Significance Statement**

Beyond the management of intraocular pressure, the importance of neuroprotective interventions in glaucoma therapy is paramount. Neuropeptide Y (NPY), an endogenous peptide known for its diverse roles, has emerged as a promising candidate for addressing neurodegenerative disorders. This study provides compelling evidence of NPY's capacity to preserve retinal integrity, modulate receptor expression, mitigate neuroinflammation, and safeguard against axonal deterioration in a glaucoma mouse model. Importantly, molecular investigations reveal that NPY exerts these protective effects predominantly through the MAPK and PI3K/Akt signaling pathways. These insights not only reinforce NPY's potential as an effective treatment for glaucoma but also enhance the understanding of its biochemical pathways, paving the way for the development of novel neuroprotective treatments.

## Introduction

Glaucoma is recognized globally as a leading cause of irreversible blindness and is characterized by the progressive loss of retinal ganglion cells (RGCs). This complex condition extends beyond the traditional hallmark of elevated intraocular pressure (IOP) and includes a range of retinal degenerative changes that intricately contribute to its pathology. Astrogliosis, or reactive gliosis, marked by the proliferation and hypertrophy of astrocytes and Müller cells, has been suggested to play a role in glaucomatous optic neuropathy (1). This reactive gliosis within the optic nerve head (ONH) and retina contributes to the remodeling of the extracellular matrix, and ONH cupping, and thereby impacts RGC survival (2). Similarly, microglial activation has been suggested to promote a chronic inflammatory response in the retinal and optic nerve tissues (3). Activated microglia release proinflammatory cytokines and contribute to oxidative stress, exacerbating RGC damage (4). Furthermore, glaucoma is associated with direct axonal injury to RGCs. This injury manifests as a disruption in axonal transport, significantly contributing to RGC apoptosis and subsequent visual field loss (5). The intricate interplay of these pathological processes underscores the multifactorial nature of glaucomatous neurodegeneration and highlights the need for comprehensive therapeutic approaches that target these diverse molecular pathways.

Neuropeptide Y (NPY) is a short 36-amino acid polypeptide, predominantly expressed and widely distributed in the mammalian central nervous system. It belongs to a group of C-terminally amidated peptides called the NPY or pancreatic polypeptide (PP) fold family, which includes peptide YY (PYY) and PP (6). They are characterized by the presence of tyrosine moieties in both ends of the molecule. NPY exerts its effects primarily through its receptor isoforms, notably Y1, Y2, Y5, and Y4 (albeit with lesser potency) (7). These receptors are G-protein-coupled and mediate diverse functions such as regulation of circadian rhythm, food intake, emotional processing, cardiovascular homeostasis, and immune function (8, 9). NPY and its receptors are also known to modulate neurotransmitter release, suggesting a key role in maintaining and regulating neuronal communication (10). NPY interaction with its receptors has been consistently shown to confer neuroprotection in various models of neuronal injury, highlighting its therapeutic potential in neuronal health and recovery (11–13). The neuroprotective role of NPY includes alleviation of glutamate excitotoxicity (mediated via Y1R, Y2R, and Y5R), reduction of ER stress (Y1R and Y2R), mitigation of oxidative stress (Y2R and Y5R), stimulation of autophagy (Y1R and Y5R), attenuation of neuroinflammation (Y1R), prevention of apoptosis and necrosis (Y2R and Y5R), and promotion of neurogenesis (Y1R, Y2R, and Y5R) (14–19).

The neuroprotective capabilities of NPY against various types of damage to retinal cells have been established through *in vitro* and *in vivo* studies (20–23). While NPY has been studied in many acute toxicity models, such as glutamate and 3,4-methylenedioxymethamphetamine toxicity, its protective effects in chronic degenerative models like glaucoma have not been explored. Given NPY's potential as a therapeutic target for various degenerative conditions, this study hypothesized that NPY administration could protect RGCs from degenerative changes in a glaucoma animal model. To test this hypothesis, the interactions of NPY with its specific receptors were investigated to understand their role in counteracting the pathophysiological processes that lead to RGC death in glaucomatous conditions. This study aimed to explore the therapeutic potential of NPY in glaucoma treatment, potentially offering a novel approach to preserving vision in individuals affected by this condition.

## Results

### Localization of NPY within the mouse retina and its altered expression under glaucoma condition

**Fig. 1.**
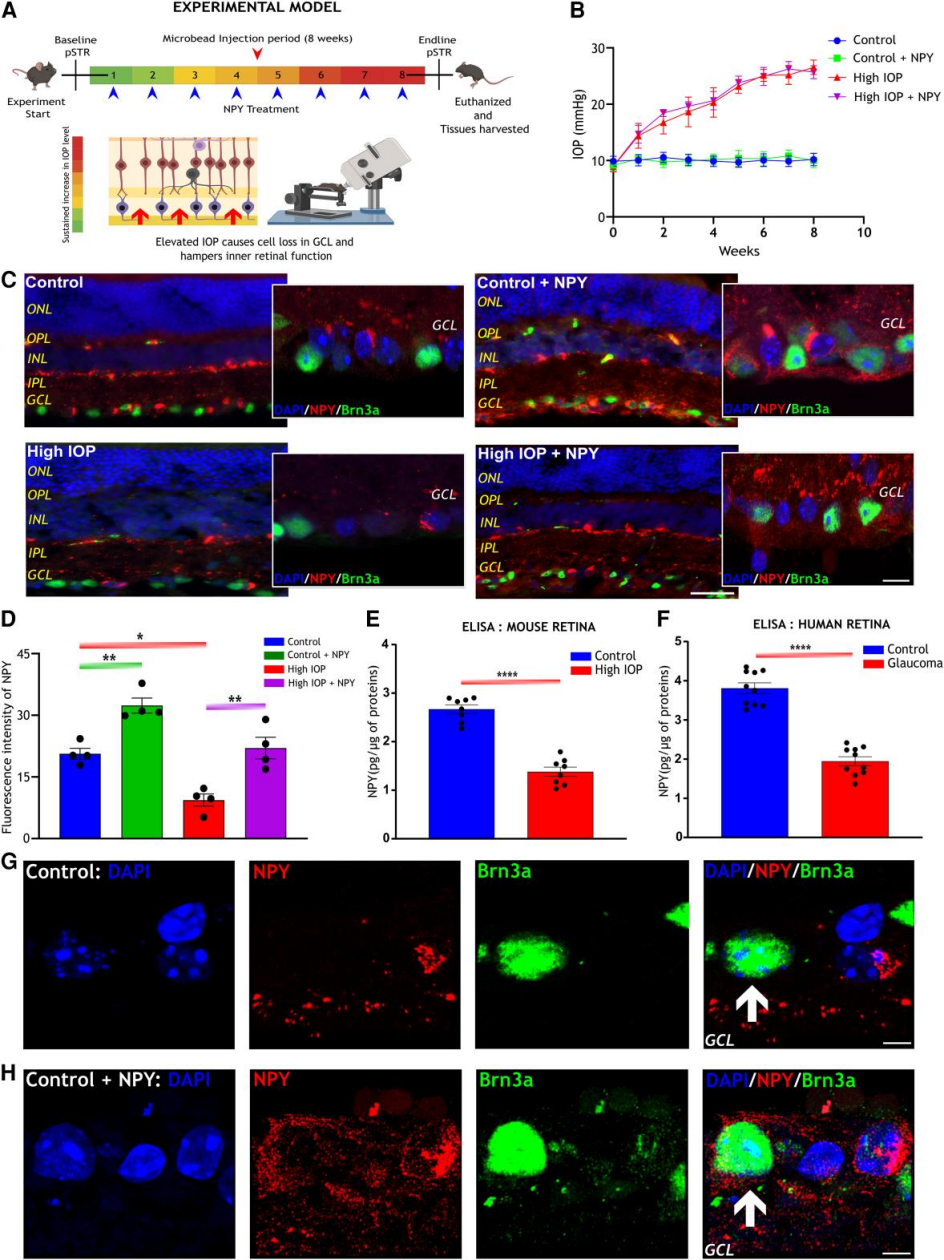
NPY expression in the mouse retina and changes in its level under high IOP conditions. A) Schematic representation of glaucoma mouse model illustrating the timeline of the experiment and highlighting the effect of elevated IOP on the inner retina. B) A graph showing the progression of IOP in control and glaucomatous mice, with and without NPY treatment over the course of 8 weeks. Immunofluorescence images of retinal sections stained with antiNPY showing its presence in INL, IPL, and GCL in both NPY-untreated (control and high IOP) and treated groups [control + NPY and high IOP + NPY (C; scale bar: 50 µm)]. Confocal microscope images show a closer look at NPY presence in the GCL (scale bar: 10 µm). D) Quantification of the fluorescence intensity of NPY indicates a significant decrease in its expression in high IOP groups compared with the control group and a notable increase in its level in NPY-treated groups (*F*(3,9) = 21.17, *P < 0.05, **P < 0.01, *n* = 4). Quantification of the ELISA revealed a significant decrease in the level of NPY in the high IOP mouse retina (*F*(7,7) = 1.208, ****P < 0.0001, *n* = 8; E) and human glaucomatous retina (*F*(9,9) = 1.450, ****P < 0.0001, *n* = 10; F) compared with the control group. H) Higher magnification confocal imaging shows the NPY expression within Brn3a-expressing RGCs in NPY-treated groups (as indicated by the arrow, scale bar: 5 µm), which contrasts with (G) depicting Brn3a-expressing RGCs in control groups (as indicated by the arrow, scale bar: 5 µm). Nuclei were stained with DAPI, and RGCs were stained with antiBrn3a. ONL, outer nuclear layer; INL, inner nuclear layer; IPL, inner plexiform layer; GCL, ganglion cell layer. Statistical significance was determined using either Student's t test for unpaired data or one-way ANOVA with Tukey's multiple comparisons test, and results are presented as mean ± SEM.

Chronic glaucomatous injury was induced in mouse eyes by intracameral microbead injection to increase IOP, and NPY treatment was delivered through intravitreal injection (Figure 1A). In comparison to the control groups (control and control + NPY), both high IOP and high IOP + NPY groups exhibited sustained increase in IOP levels (control, 10.09 ± 1.17; microbead, 26.17 ± 1.28 mmHg) (Figure 1B). In the mouse retina, NPY expression was previously identified mainly in two layers, the innermost row of the inner nuclear layer (INL) and the ganglion cell layer (GCL) (5, 21). The changes in the expression patterns of endogenous NPY under elevated IOP conditions within the retina were investigated through immunohistochemical analysis. Sagittally sectioned whole eye tissues were immunostained with antiNPY along with antiBrn3a, a marker of RGC to assess the NPY expression within these cells. Immunofluorescence staining exhibited a notable NPY immunoreactivity in the innermost row of INL, inner plexiform layer (IPL), and a subset of cells within the GCL (Figure 1C). However, confocal imaging of the cells in GCL revealed that NPY immunoreactivity did not exactly colocalize with Brn3a-expressing RGCs in both the control and high IOP groups. Quantitative analysis demonstrated a significant decrease in NPY immunoreactivity levels in the high IOP group compared with the controls (*P* < 0.05; Figure 1D). Specifically, a distinct decrease in its immunoreactivity was observed in the GCL, suggesting a potential correlation between NPY levels and the pathogenesis of glaucoma. ELISA tests on retinal samples further confirmed significantly lower NPY levels in both human glaucomatous retinas and mouse models of induced glaucoma. The concentrations of NPY were found to be 3.81 ± 0.43 pg/µg in human and 2.67 ± 0.24 pg/µg in mouse retina samples and these levels were observed to be significantly reduced to 1.94 ± 0.36 and 1.38 ± 0.26 pg/µg, respectively, in glaucomatous conditions (*P* < 0.0001; Figure 1E and F). In the mice groups treated with exogenous NPY via intravitreal injection (both control + NPY and high IOP + NPY), there was a notable elevation in the NPY immunoreactivity (*P* < 0.01; Figure 1D). This increase was significant when compared with the control groups that did not receive NPY treatment (control and high IOP). Upon closer investigation through high-resolution confocal imaging, prominent NPY immunoreactivity was observed within Brn3a-expressing RGCs in NPY-treated groups (as indicated by the white arrow, Figure 1H). This observation contrasted with the Brn3a-expressing RGCs in untreated control groups (white arrow, Figure 1G). These results indicated that the levels of NPY in mouse retina were altered under high IOP conditions, hinting at potential association with glaucoma. Additionally, the NPY immunoreactivity in Brn3a-expressing RGCs of NPY-treated groups suggested a possible internalization of the exogenous NPY into the RGCs.

### NPY receptor distribution and their expression level in glaucomatous mouse retina

**Fig. 2.**
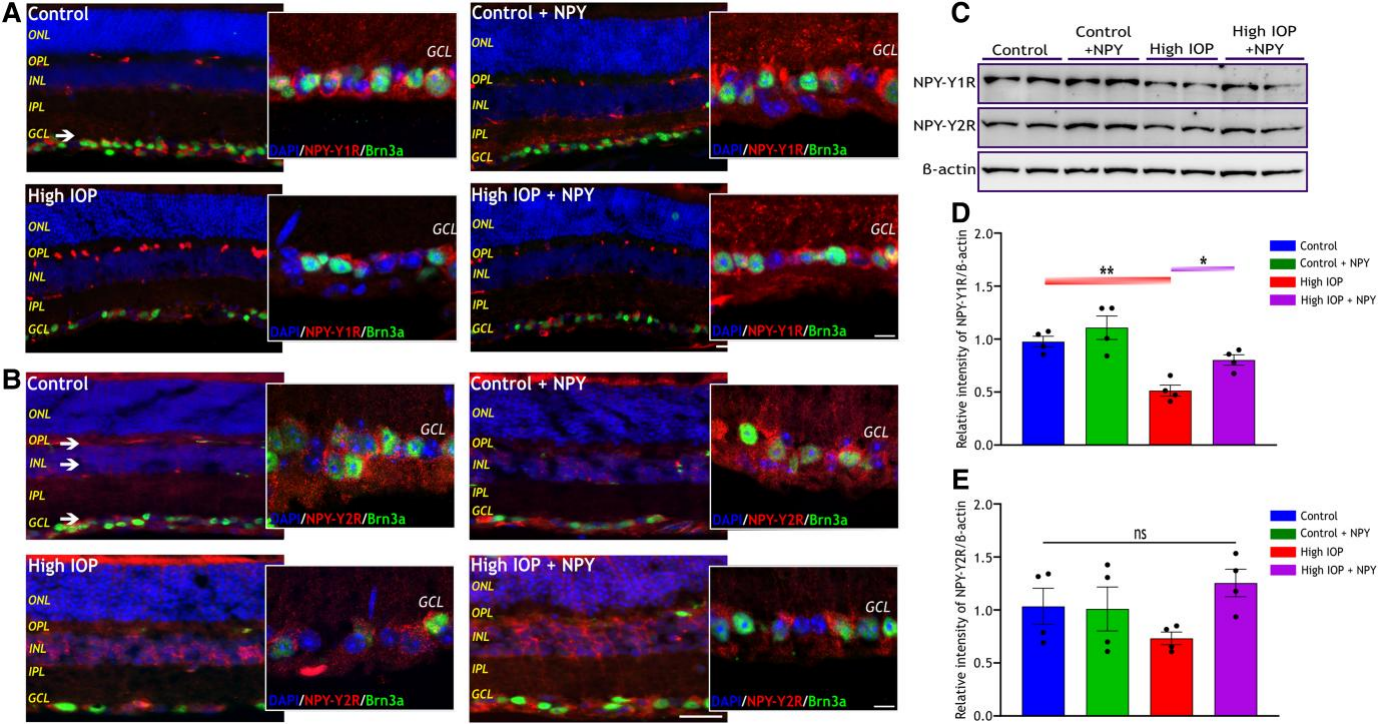
Distribution of NPY-Y1 and Y2 receptors within the mouse retina and altered expression under high IOP conditions. Representative immunofluorescence images of the retina stained with antiNPY-Y1R (A) and antiNPY-Y2R (B), showing their presence in various retina layers (as indicated by the arrow, scale bar: 50 µm). Confocal imaging was carried out to take a closer look at the NPY receptor's presence in Brn3a-expressing RGCs in the GCL (scale bar: 10 µm). Nuclei were stained with DAPI, and RGCs were stained with antiBrn3a. ONL, outer nuclear layer; OPL, outer plexiform layer; INL, inner nuclear layer; IPL, inner plexiform layer; GCL, ganglion cell layer. C) Western immunoblot analysis of the level of NPY-Y1R and NPY-Y2R in both control (control and control + NPY) and high IOP groups (high IOP and high IOP + NPY). Densitometric quantification of NPY-Y1R (D) reveals a significant decrease in its level in high IOP groups and an increase with NPY treatment (*F*(3, 12) = 13.18, *P < 0.05, **P < 0.01, *n* = 4). Densitometric quantification of NPY-Y2R (E) reveals no significant change in its level within groups (*F*(3, 12) = 1.979, ns represents nonsignificance, *n* = 4). Each band intensity was normalized to the respective band intensity of β-actin. Statistical significance was determined using a one-way ANOVA with Tukey's multiple comparisons test, and results are presented as mean ± SEM.

**Fig. 3.**
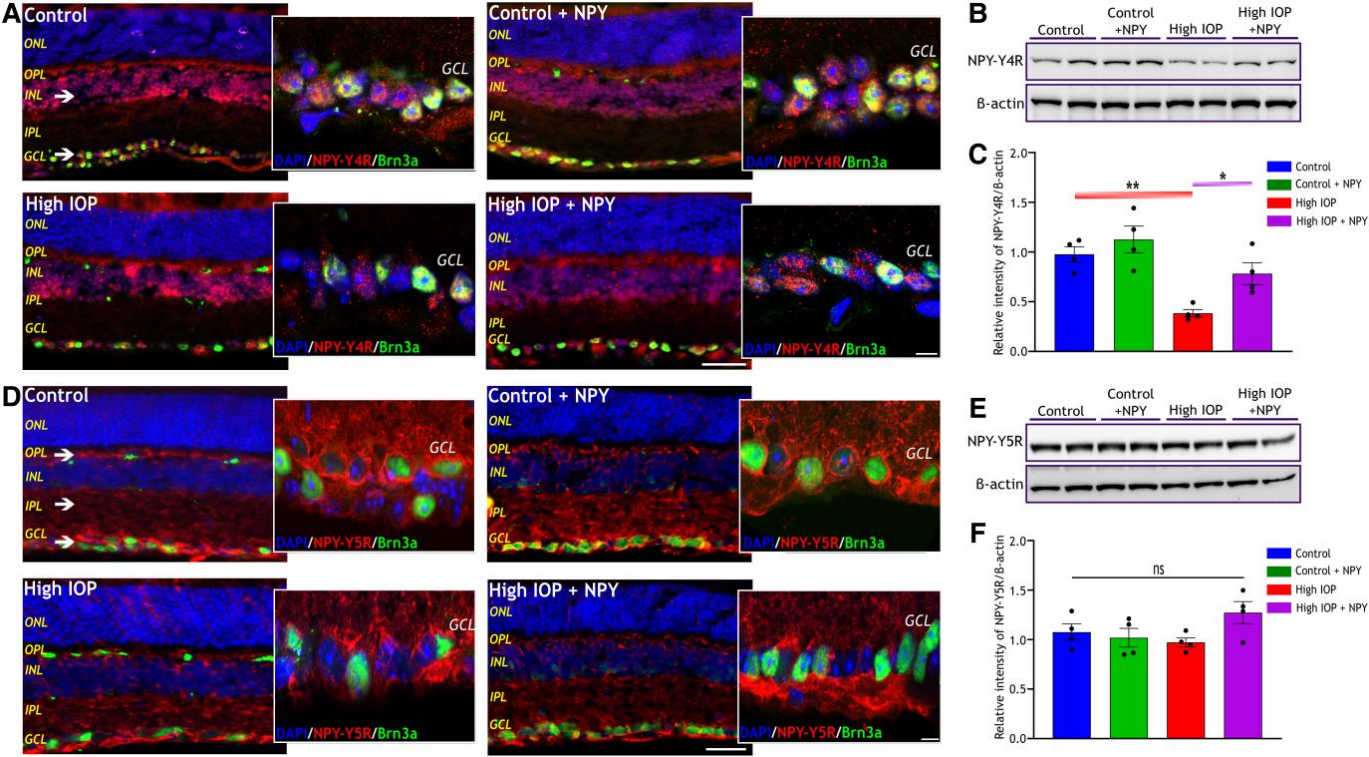
Distribution of NPY-Y4 and Y5 receptors within the mouse retina and altered expression under high IOP conditions. Representative immunofluorescence images of the retina stained with antiNPY-Y4R (A) and antiNPY-Y5R (D), showing their presence in various retina layers (as indicated by the arrow, scale bar: 50 µm). Confocal imaging was carried out to take a closer look at the NPY receptor's presence in Brn3a-expressing RGCs in the GCL (scale bar: 10 µm). Nuclei were stained with DAPI, and RGCs were stained with antiBrn3a. ONL, outer nuclear layer; OPL, outer plexiform layer; INL, inner nuclear layer; IPL, inner plexiform layer; GCL, ganglion cell layer. Western immunoblot analysis of NPY-Y4R (B) and its densitometric quantification (C) reveals a significant decrease in its level in high IOP groups and an increase with NPY treatment (*F*(3, 12) = 11, *P < 0.05, **P < 0.01, *n* = 4). Western immunoblot analysis of NPY-Y5R (E) and its densitometric quantification (F) reveals no significant change in its level within groups [*F*(3, 12) = 2.318, ns represents nonsignificance, *n* = 4]. Each band intensity was normalized to the respective band intensity of β-actin. Statistical significance was determined using a one-way ANOVA with Tukey's multiple comparisons test, and results are presented as mean ± SEM.

Numerous studies have highlighted that NPY influences its effects primarily through NPY-Y1, Y2, Y4, and Y5 receptors, and their expression in different layers of the retina across various species (20, 22, 24). The distribution pattern and alterations in the expression levels of NPY receptors in the mouse retina under glaucomatous conditions were assessed using immunofluorescence and western blotting analyzes. Immunostaining of whole eye sections with NPY receptor antibodies (Y1R, Y2R, Y4R, and Y5Rs), unveiled a distinctive localization pattern of these receptors within the mouse retinal layers (Figures 2A and B, 3A and D). NPY-Y1R immunoreactivity was predominantly detected in cells within the GCL across all groups (indicated by the white arrow in Figure 2A), while Y2R, Y4R, and Y5R immunoreactivity were observed in the outer plexiform layer (OPL), INL, and GCL (indicated by the white arrow in Figures 2B, 3A and D, respectively). Co-staining of retinal sections with antiBrn3a demonstrated a significant expression of all NPY receptors within the RGC and other nonBrn3a-expressing cells in GCL. This was ascertained by the higher magnification (scale: 10 µm) images of GCL taken through confocal imaging. The level of NPY receptors within the control groups (control and control + NPY) and high IOP groups (high IOP and high IOP + NPY) was evaluated by western blot analysis of retinal lysates. Upon quantitative analysis of western blot band intensities, a significant decrease in the NPY-Y1R and Y4R levels was observed in the high IOP group compared with the control (*P* < 0.01, Figures 2D and 3C). Intriguingly, treatment with exogenous NPY significantly prevented their downregulation (*P* < 0.05, Figures 2D and 3C), suggesting a potential modulatory effect of NPY on the expression of these receptor subtypes under glaucomatous conditions. However, no significant changes in the levels of NPY-Y2R and Y5R were observed in response to high IOP conditions and NPY-treated groups (Figures 2E and 3F). These findings indicate an intricate involvement of NPY receptors in the pathophysiology of glaucoma, particularly their distinct modulation in response to elevated IOP.

### NPY treatment imparted protection against structural and functional loss of the inner retina in glaucoma conditions

**Fig. 4.**
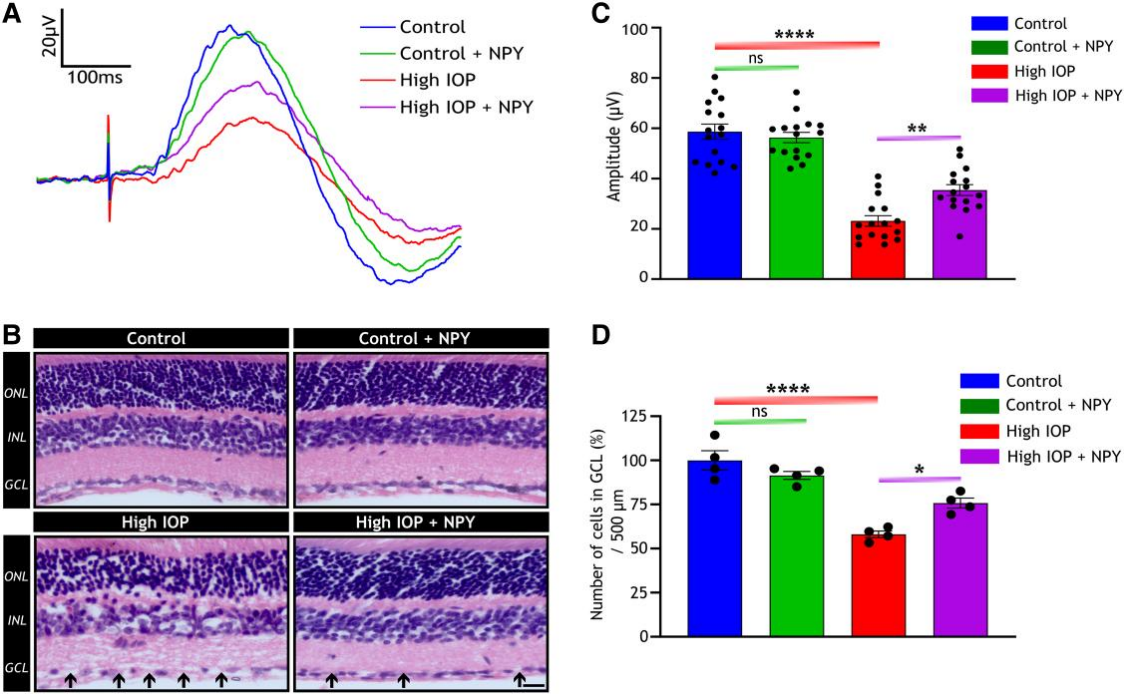
Protective effect of NPY treatment in preserving the function and structure of the inner retina under high IOP conditions. A) Representative image of pSTR amplitudes recorded after 8 weeks of treatment and its quantification (B) reveals a significant decrease in amplitude under high IOP conditions and a subsequent increase following NPY treatment [*F*(3,60) = 52.19, ns represents nonsignificance, **P < 0.01, ****P < 0.0001, *n* = 16]. C) Representative images of H&E-stained cross-section of the retina, where black arrows indicate cell loss (scale bar: 25 µm). ONL, outer nuclear layer; INL, inner nuclear layer; GCL, ganglion cell layer. D) Quantification of the H&E staining reveals a significant cell loss within GCL in high IOP groups compared with control and an increase in cell count with NPY treatment [*F*(3, 9) = 35.47, ns represents nonsignificance, *P < 0.05, ****P < 0.0001, *n* = 4]. Statistical significance was determined using a one-way ANOVA with Tukey's multiple comparisons test and results are presented as mean ± SEM.

The effects of NPY treatment on the structural and functional integrity of the mouse retina under conditions of elevated IOP were further investigated. Previous studies have demonstrated that elevated IOP exposure over a period of time using microbeads can lead to optic nerve damage, dysfunction of the inner retina, and cell loss (25). To assess glaucomatous changes, positive scotopic threshold response (pSTR) amplitudes that reflect the inner retinal function were measured, and hematoxylin and eosin (H&E) staining of retinal sections was performed for laminar structural analysis. The pSTR measurements showed a significant reduction in amplitude in the high IOP group as compared with the control (*P* < 0.0001, Figure 4A and B). This reduction is indicative of the detrimental impact of elevated IOP on inner retinal function. In the high IOP group treated with NPY, there was a significant preservation of pSTR amplitude, suggesting that NPY treatment mitigated the functional impairment caused by elevated IOP (*P* < 0.01, Figure 4A and B).

In terms of structural integrity, H&E staining of retinal sections revealed notable cell loss under high IOP conditions within the GCL, as marked by the black arrow in Figure 4C. This loss was quantitatively significant compared with the control group (*P* < 0.0001, Figure 4D). However, with NPY treatment, this loss was reduced, showing the protective effects against cell loss under high IOP conditions (*P* < 0.05, Figure 4D). Importantly, no significant alterations in pSTR amplitude or cell loss in GCL were observed in the control group treated with NPY, indicating that NPY treatment did not exert any toxic effects on retinal cells. Together, these findings underscore the potential of NPY treatment in preserving both the structural and functional integrity of the inner retina under elevated IOP conditions.

### NPY mitigates glial cell activation in the glaucomatous mouse retina

**Fig. 5.**
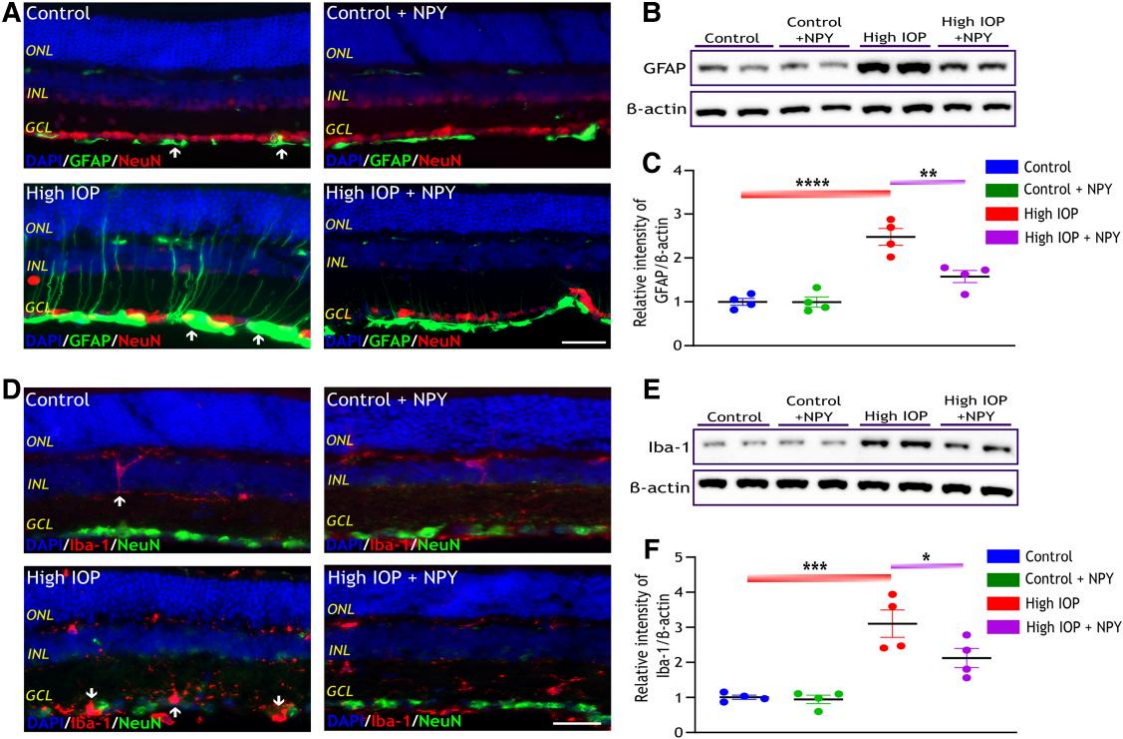
NPY alleviates astroglial damage and reduces microglial activation in the glaucomatous mouse retina. A) Representative immunofluorescence images of retina stained with GFAP antibody to highlight astrocytes and NeuN antibody to highlight neuronal cells, scale bar: 50 µm. White arrows indicate areas with heightened GFAP expression. Western blotting (B) and the densitometric analysis of GFAP expression (C) reveal a substantial increase in the high IOP group relative to the control and a significant decrease in expression with NPY treatment even under high IOP conditions [*F*(3,12) = 25.85, **P < 0.01, ****P < 0.0001, *n* = 4]. D) Representative immunofluorescence image of retina stained with Iba-1 antibody to highlight microglia and NeuN antibody to highlight neuronal cells, scale bar: 50 µm. White arrows indicate areas with heightened Iba-1 expression. Western blotting (E) and the densitometric analysis of Iba-1 expression (F) across all groups reveals a substantial increase in the high IOP group relative to the control and a significant decrease in expression with NPY treatment even under high IOP conditions [*F*(3,12) = 17.23, *P < 0.05, ***P < 0.001, *n* = 4]. Each band intensity was normalized to the respective band intensity of β-actin. Statistical significance was determined using a one-way ANOVA with Tukey's multiple comparisons test (mean ± SEM). Nuclei were stained with DAPI. ONL, outer nuclear layer; INL, inner nuclear layer; GCL, ganglion cell layer.

Previous research has indicated that injury caused by IOP elevation in the retina may trigger activation of resident microglia and macroglia (astrocytes and Müller cells) (3). This hyperactivation is generally believed to adversely affect the RGC integrity. Reactive astrogliosis is characterized by an elevated expression of glial fibrillary acidic protein (GFAP), while the activation of microglia is denoted by enhanced expression of ionized calcium-binding adaptor molecule 1 (Iba-1) (26, 27). To evaluate the potential neuroprotective effects of NPY on the activation of macroglia and microglia in the retina, an analysis of GFAP and Iba-1 expression was conducted using immunofluorescence and western blot techniques. Immunofluorescence staining of retinal cross-sections revealed a marked GFAP and Iba-1 upregulation in GCL under high IOP conditions compared with the control group, as illustrated in Figure 5A and D. Additionally, the sections were co-stained with antiNeuN to explore the interactions between glial and neuronal cells. In high IOP retinas, an extensive network of processes from astrocytes and Müller cells was observed, indicative of the hypertrophic state characteristic of reactive astrogliosis (as indicated by the white arrow in Figure 5A). Similarly, microglial cells expressing Iba-1 were observed in proximity to neuronal cells, with notable alterations in their morphology (as indicated by the white arrow in Figure 5D).

Western blot analysis of the retina and its densitometric quantification also revealed a significant elevation in GFAP expression in high IOP conditions compared with the control group (*P* < 0.0001, Figure 5B and C). Notably, this increase was significantly mitigated following treatment with NPY (*P* < 0.01, Figure 5B and C). Similarly, quantification of Iba-1 western blot intensities demonstrated a significant increase in its expression under high IOP conditions (*P* < 0.001), which was attenuated by NPY treatment (*P* < 0.05, Figure 5E and F). Conversely, in groups solely treated with NPY, no remarkable changes were observed in the expression of GFAP and Iba-1 (Figure 5C and F). These findings suggest that NPY treatment is effective in reducing reactive gliosis and microglial activation in high IOP glaucomatous conditions.

### Effect of NPY treatment on axonal damage and other molecular changes in optic nerve under high IOP conditions

**Fig. 6.**
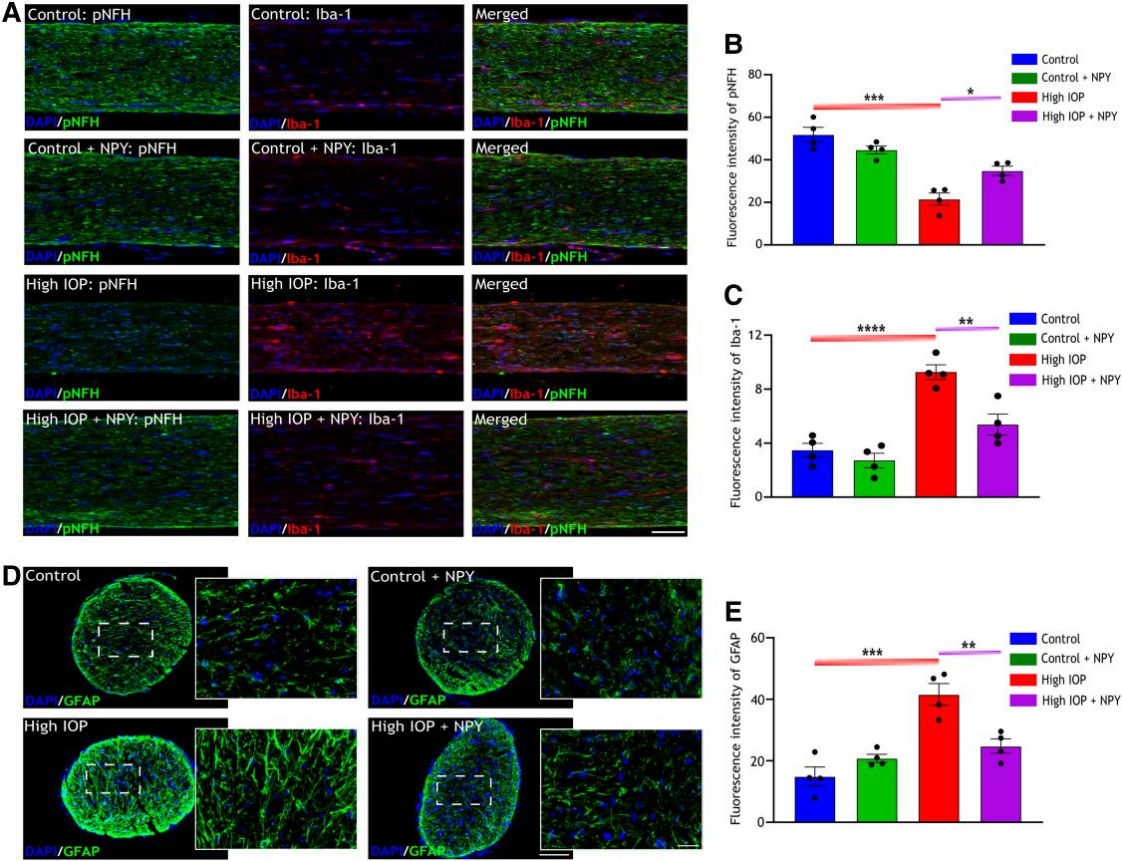
NPY reduces axonal injury and degenerative alterations in the optic nerve under glaucomatous conditions. A) Representative immunofluorescence images of optic nerve longitudinal sections stained with pNFH antibody and Iba-1 antibody (highlighting microglia), scale bar: 50 µm. B) The fluorescence intensity quantification of pNFH indicates a decrease in the high IOP group compared with the control, with a significant elevation in its level following NPY treatment in high IOP conditions [*F*(3,9) = 19.36, *P < 0.05, ***P < 0.001, *n* = 4]. C) Quantification of Iba-1 fluorescence intensity shows a notable increase in the high IOP group when compared with the control, with a significant reduction in its expression after NPY treatment in high IOP conditions [*F*(3,12) = 23.61, **P < 0.01, ***P < 0.001, *n* = 4]. D) Representative immunofluorescence images of optic nerve cross-sections stained with GFAP antibody highlighting the astrocytes, with a scale bar of 50 µm and higher magnification images featuring a scale bar of 10 µm. E) Quantification of GFAP fluorescence intensity indicates a significant increase in the high IOP group compared with the control, with a notable decrease in expression following NPY treatment under high IOP conditions [*F*(3,9) = 19.13, **P < 0.01, ***P < 0.001, *n* = 4]. Nuclei were stained with DAPI. Statistical significance was determined using a one-way ANOVA with Tukey's multiple comparisons test (mean ± SEM).

The effects of elevated IOP and NPY treatment on RGC axons were further explored by assessing the integrity of neurofilament heavy chain (NFH) in the optic nerve. The neurofilaments are crucial components of the cytoskeleton, playing a key role in maintaining axonal structure and function, particularly, changes in the phosphorylated forms of NFH serve as biomarkers often associated with neurodegeneration (28–30). Therefore, immunofluorescence staining of optic nerve longitudinal sections was conducted using antibodies against phosphorylated neurofilament heavy chain (pNFH) and Iba-1 (highlighting microglia), as these markers exhibit contrasting levels of expression under degenerative conditions. Immunofluorescence imaging of optic nerves under high IOP conditions displayed a marked reduction in pNFH immunoreactivity and an increase in Iba-1 expression, compared with the control group (Figure 6A). The quantified fluorescence intensity of pNFH indicated a significant decrease in its levels in the high IOP group (*P* < 0.001), which was effectively protected upon NPY treatment (*P* < 0.05, Figure 6B). In parallel, the fluorescence intensity quantification of Iba-1 demonstrated a substantial elevation under high IOP conditions (*P* < 0.001, Figure 6B), which was significantly reduced with NPY treatment (*P* < 0.01, Figure 6C). Notably, in groups treated solely with NPY, there were no alterations in the levels of pNFH and Iba-1, when compared with the control group (Figure 6B and C).

In addition to the previous analyzes, astroglial reactivity in optic nerve injury was investigated. Immunofluorescence imaging of optic nerve cross-sections stained with the GFAP antibody showed a notable increase in its expression under conditions of elevated IOP (Figure 6D). Higher magnification images further highlighted the hypertrophic characteristics of astrocytes in the high IOP group, in comparison to the controls (Figure 6D). Quantitative analysis of these images demonstrated a significant elevation in GFAP levels under high IOP conditions (*P* < 0.001), which was effectively mitigated by treatment with NPY (*P* < 0.01, Figure 6E). These findings are consistent with the previous results in retinal tissue, where NPY treatment exhibited neuroprotective effects, not only by maintaining axonal integrity and modulating microglial activation but also by reducing astroglial injury.

### Role of MAPK and phosphatidylinositol-3 kinase/Akt signaling in mediating NPY protective effects in a glaucomatous mouse retina

**Fig. 7.**
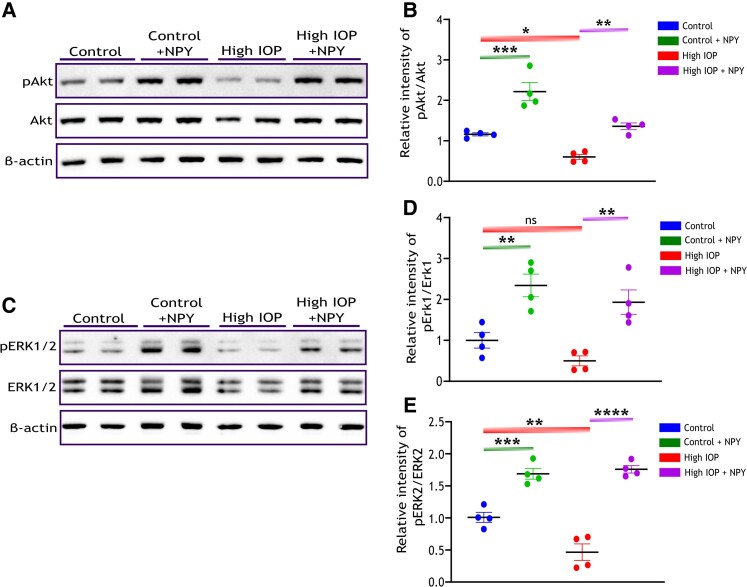
NPY treatment upregulates the phosphorylation of ERK1/2 and Akt under high IOP conditions. A) Western immunoblot analysis and its quantification (B) of Akt phosphorylation (Ser473) reveal a significant decrease in its level under high IOP conditions compared with the control, and NPY treatment significantly increased its level [*F*(3,12) = 29.20, *P < 0.05, **P < 0.01, ***P < 0.001, *n* = 4]. C) Western immunoblot analysis and its quantification of ERK1 (D) and ERK2 (E) phosphorylation (Thr202/Tyr204) reveal a significant decrease in its level under high IOP conditions (ERK2 only) compared with the control, and NPY treatment significantly increased its level [*F*(3,12) = 13.17 in (D) and *F*(3,12) = 44.90 in (E), **P < 0.01, ***P < 0.001, ****P < 0.0001, *n* = 4]. Phosphorylated Akt and ERK1/2 intensity were normalized to the respective band intensity of total Akt and ERK1/2, respectively. Statistical significance was determined using a one-way ANOVA with Tukey's multiple comparisons test (mean ± SEM).

Previous studies have highlighted that the NPY mediates its neuroprotective effects primarily through the phosphorylation and activation of mitogen-activated protein kinase (MAPK) (ERK1/2) and Phosphatidylinositol-3 kinase (PI3K)/Akt pathways (13, 31). PI3K/Akt/mTOR and ERK1/2 signaling play a critical role in neuronal survival, underscoring their importance in neuroprotective mechanisms (32). Thus, the phosphorylation levels of Akt (Ser473) and ERK1/2 (Thr202/Tyr204) in retinal lysates were investigated by western blot analysis. Densitometric quantification of western blots revealed a significant decrease in Akt phosphorylation levels in the retina subjected to high IOP (*P* < 0.05, Figure 7A and B). Interestingly, this decrease was specific to the phosphorylation level of ERK2 in the high IOP groups (*P* < 0.01, Figure 7C–E), with no significant change observed in ERK1. NPY treatment led to a significant increase in the phosphorylation levels of both Akt (*P* < 0.01, Figure 7A and B) and ERK1/2 (*P* < 0.01 for ERK1 and *P* < 0.0001 for ERK2, Figure 7C–E) in high IOP conditions. Additionally, a significant elevation in phosphorylation levels of ERK1/2 was observed in the group solely treated with NPY (*P* < 0.01 in ERK1, *P* < 0.001 in ERK2, Figure 7C–E).

**Fig. 8.**
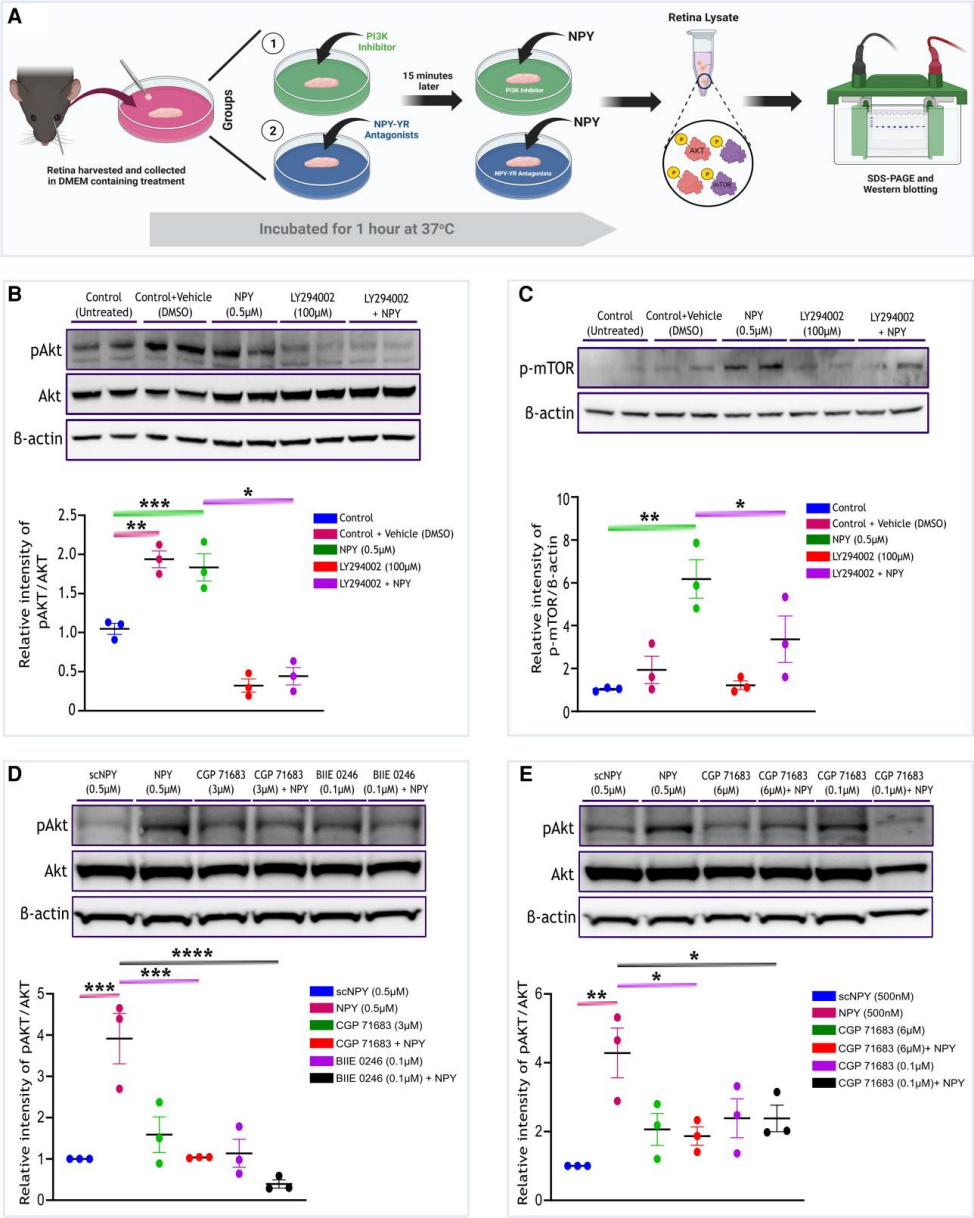
NPY exerts its influence on the mouse retina by modulating the PI3K/Akt signaling pathways. A) Schematic representation of workflow of ex vivo PI3K inhibitor and NPY receptor antagonist treatment in the presence and absence of NPY (created with Biorender.com). Western immunoblot and its quantification of Akt (Ser473) (B) and mTOR (Ser2448) (C) phosphorylation reveal a significant increase in its level in the retina treated with NPY only and significant inhibition of its phosphorylation in the group exposed to LY2940002 and NPY [*F*(4, 8) = 41.86 in (B) and *F*(4, 8) = 21.32 in (C), *P < 0.05, **P < 0.01, ***P < 0.001, *n* = 3]. D) Western immunoblot and its quantification of Akt phosphorylation (Ser473) reveal a significant increase in its level in the retina treated with NPY only and significant inhibition of its phosphorylation in the group exposed to CGP 7183 (3 µM) and BIIE 0246 (0.1 µM) with NPY [*F*(5, 12) = 13.42, ***P < 0.001, ****P < 0.0001, *n* = 3]. E) Western immunoblot and its quantification of Akt phosphorylation (Ser473) reveal a significant increase in its level in the retina treated with NPY only and significant inhibition of its phosphorylation in the group exposed to CGP 7183 (6 µM) and CGP 71683 (0.1 µM) with NPY [*F*(5, 12) = 5.517, *P < 0.05, **P < 0.01, *n* = 3]. The phosphorylated Akt intensity was normalized to the respective band intensity of total Akt. Phosphorylated mTOR intensity was normalized to the respective band intensity of β-actin. Statistical significance was determined using a one-way ANOVA with Tukey's multiple comparisons test (mean ± SEM).

Furthermore, to gain deeper insights into the signaling mechanism of NPY-mediated activation of the PI3K/Akt/mTOR pathway, an ex vivo experiment was conducted involving the PI3K inhibitor LY294002 (100 µM). This inhibitor was employed to block PI3K signaling in the retina (Figure 8A). Western blot analysis revealed a significant upregulation in the phosphorylation levels of Akt (Ser473) (*P* < 0.001, Figure 8B) and mTOR (Ser2448) (*P* < 0.01, Figure 8C) in the retina treated with only NPY (0.5 µM). However, in retinas treated with both the PI3K inhibitor LY294002 and NPY, a decreased level of phosphorylation of Akt (Ser473) (*P* < 0.05, Figure 8B) and mTOR (Ser2448) (*P* < 0.01, Figure 8C) was observed compared with retinas treated with NPY alone. Interestingly, a significant upregulation of Akt phosphorylation level (Ser473) was observed in the retina treated with dimethylsulphoxide (DMSO) (*P* < 0.001, Figure 8B), compared with the control. This effect was possibly caused by the DMSO-induced activation of Akt as explained in previous studies (33).

To further examine the activation mechanisms of Akt by NPY, a series of pharmacological experiments were conducted to determine the involvement of different NPY receptors. Specific antagonists for each NPY receptor subtype were utilized: Y1R antagonist CGP 71683 at 3 µM, Y2R antagonist BIIE 0246 at 0.1 µM, Y4R antagonist CGP 71683 at 6 µM, and Y5R antagonist CGP 71683 at 0.1 µM. These antagonists were incubated with the retina both in the presence and absence of NPY (Figure 8A). The impact of these receptor-specific antagonists on Akt phosphorylation was then assessed through western blot analysis of retinal lysates. Quantification of the western blots revealed a significant increase in phosphorylation levels of Akt (Ser473) in NPY (*P* < 0.001, Figure 8D and E) treated conditions compared with the retinas treated with scrambled control NPY peptide (scNPY). However, in retinas exposed to NPY receptor antagonists and NPY, a decreased level of phosphorylation of Akt (Ser473) was observed (*P* < 0.001 for CGP 71683 at 3 µM, *P* < 0.0001 for BIIE 0246 at 0.1 µM, *P* < 0.001 for CGP 71683 at 6 µM, and *P* < 0.05 for CGP 71683 at 0.1 µM, Figure 8D and E). These results help characterize the role of different NPY receptors in activating the PI3K/Akt signaling pathway, further emphasizing the intricate nature of NPY's neuroprotective mechanisms.

## Discussion

NPY plays a complex role in several physiological processes and has been suggested as a potential therapeutic agent to modulate neurodegenerative pathways in various disorders (14, 34). This study investigated the role of NPY and its receptors in glaucoma and revealed neuroprotective ability in countering retinal degenerative changes caused by elevated IOP in the mouse model. The initial investigations were focused on the expression of endogenous NPY in the mouse retina, especially within RGCs. The results corroborated previous observation reports, highlighting that NPY is primarily localized in cells within the INL, IPL, and GCL, predominantly in amacrine and displaced amacrine cells of the mouse retina (20, 21, 35). Interestingly, no significant colocalization of endogenous NPY immunoreactivity was evident in Brn3a-expressing RGCs as shown in Figure 1G. Following this observation, the focus was directed toward examining the expression of NPY receptors—specifically Y1R, Y2R, Y4R, and Y5R—within the RGCs.

Consistent with earlier discoveries, the expression of NPY receptors was detected in various layers of the retina, including in Brn3a-expressing RGCs (20–22). The absence of endogenous NPY immunoreactivity in Brn3a-positive RGCs suggested a more complex mechanism of NPY action in the retina than previously understood. Based on these findings, it is proposed that NPY likely exerts its effects on RGCs through a paracrine mechanism. This process may be influenced by NPY produced in amacrine and displaced amacrine cells and subsequent interaction with receptors on RGCs. Additionally, the impact of NPY treatment on Brn3a-expressing RGCs is noteworthy. The presence of exogenous NPY within RGCs was observed in the NPY-treated groups, likely occurring via receptor-mediated internalization, as indicated in Figure 1H. Previous studies have specifically implicated the NPY-Y1 receptor in facilitating this internalization of its agonist (36, 37). This finding suggests that RGCs in the mouse retina may be capable of taking up externally administered NPY.

As a novel finding, this study has identified changes in the expression levels of endogenous NPY and a subset of its receptors under retinal degenerative conditions induced by elevated IOP. A decreased NPY expression was observed under glaucomatous conditions both in mouse and human retinas (Figure 1). This reduction mirrors the observations made in other neurodegenerative diseases, such as Alzheimer's disease which shows molecular overlap of pathological processes with glaucoma (38–40). The findings also revealed a significant decrease in the levels of NPY-Y1R and NPY-Y4R in response to elevated IOP, while NPY-Y2R and NPY-Y5R remained unaffected (Figures 2 and 3). The results obtained from competitive ELISA showed a relatively higher baseline expression of NPY-Y2R and NPY-Y5R (Figure S1) in the mouse retina compared with other receptors. This aligns with previous results, suggesting that the widespread expression of these receptors contributes to their unaffected levels under high IOP conditions. Notably, exogenous NPY treatment restored NPY1R and NPY4R levels, suggesting mitigation of the degradative changes induced by high IOP. Although the precise mechanism of this upregulation is not fully understood, previous research has shown that NPY receptor activation can initiate its receptor gene expression through the activation of CaM kinase-CREB pathway (41–43). Furthermore, research has demonstrated overlapping NPY-Y1R and Y5R genes, likely from a gene duplication event, indicating coordinated expression when stimulated (44). This coordinated expression could be a factor in the regulation of the receptors following NPY treatment.

A significant finding of this study is the ability of NPY to mediate the preservation of inner retinal functional and structural integrity under elevated IOP conditions, as demonstrated in Figure 4. The pSTR amplitude is a critical indicator of RGC functionality and overall inner retinal health. The relative preservation of pSTR amplitude despite exposure to elevated IOP suggests the crucial role of NPY in maintaining retinal functional integrity. Additionally, the study highlighted NPY's role in promoting cell survival demonstrated by its ability to prevent cell loss within the GCL in the context of high IOP. Furthermore, the findings suggest that NPY treatment can protect against axonal damage in the optic nerve, a hallmark of glaucomatous degeneration (Figure 6). It has been widely reported that the dephosphorylation of NFH disrupts axonal transport, neuronal plasticity, and morphology in disease conditions (29, 45, 46). Therefore, safeguarding these axons is crucial for preserving vision in individuals with glaucoma. NPY's effectiveness in reducing the dephosphorylation of NFH under high IOP conditions underscores its comprehensive neuroprotective role.

Glaucoma is accompanied by reactive gliosis and microglial activation, contributing to a neuroinflammatory environment that can further exacerbate retinal neuronal loss. In glaucomatous conditions, morphological changes, differentiation, and upregulation of GFAP in macroglia (astrocytes and Müller cells) and Iba-1 in microglia are indicative of the degenerative stress inflicted upon the retina. NPY has previously been shown to contribute to the mitigation of neuroinflammation across various neurodegenerative diseases, primarily through its activation of specific receptors on glial cells (11, 47, 48). Predominantly, NPY is known to activate the Y1 receptor on glial cells, which plays a pivotal role in inhibiting their proinflammatory responses, thus effectively counteracting the neuroinflammatory process (49). This is corroborated by the observation of reduced levels of reactive astrogliosis markers (GFAP) and microglial activation (Iba-1) following NPY treatment in the high IOP group (Figure 5). Additionally, the data indicated a possible link between the decreased expression of NPY-Y1R under high IOP conditions and the enhanced activation of glial cells typically seen in glaucoma. A similar downregulation of GFAP and Iba-1 expression was evident in the optic nerves of the high IOP + NPY group, further substantiating the extensive protective effect of NPY along the visual pathway (Figure 6). This comprehensive impact of NPY, mitigating both retinal and optic nerve inflammation, reinforces its potential as a therapeutic agent in glaucoma treatment.

The previous work involving SH-SY5Y cell lines demonstrated a dose-dependent activation of ERK1/2 and Akt upon treatment with NPY alone (50). These observations prompted a more detailed examination of these prosurvival pathways in the context of high IOP and NPY-treated retinas in vivo. The phosphorylation levels of Akt (Ser473) and ERK1/2 (T202/Y204), which were diminished under high IOP conditions, were notably upregulated following NPY administration (Figure 7). This suggests that NPY exerts a protective effect by possibly counteracting the stress-induced downregulation of these crucial survival pathways. Furthermore, a significant elevation in ERK1/2 phosphorylation was observed in retinas treated exclusively with NPY, aligning with the earlier findings. While numerous studies have documented NPY's modulation of the ERK1/2 and p38 pathways, evidence regarding its influence on PI3K/Akt pathways has been sparse (11, 51, 52).

This study also explored the involvement of NPY in the PI3K/Akt signaling pathway, which is crucial for cell survival and metabolism, particularly in the context of glaucoma. The experiment involving the PI3K inhibitor LY294002 provided valuable insights into the specific role of the PI3K/Akt pathway in NPY-mediated signaling (Figure 8A). The inhibition of PI3K activity using LY294002 resulted in a notable reduction in the activation of downstream signaling components, Akt and mTOR, in response to NPY treatment (Figure 8B and C). These results demonstrated the modulation of the PI3K/Akt/mTOR pathway by NPY. Further elucidating the mechanism of NPY's action, the experiments employing specific antagonists for NPY receptors (Y1R, Y2R, Y4R, and Y5R) revealed that Akt phosphorylation in response to NPY is dependent on the activation of these receptors. The observed decrease in Akt phosphorylation upon treatment of these receptor-specific antagonists, even in the presence of NPY (as shown in Figure 8D and E), emphasized that NPY neuroprotective actions were receptor-dependent. Additionally, competitive ELISA results, as shown in Figure S1, indicated the interaction of NPY and its receptors in the retina, further supporting the potential of targeting specific NPY receptors to modulate the PI3K/Akt pathway.

NPY receptors belong to the Class A rhodopsin-like G-protein-coupled receptor (GPCRs) family, which are generally coupled to G_i_ and G_o_ proteins (7). The activation of the GPCR-mediated PI3K/Akt pathway is a recognized downstream event and a promising therapeutic target in neurodegenerative diseases such as Parkinson's (53) and Alzheimer's diseases (54). This connection highlights the therapeutic potential of NPY and its receptors in the field of neurodegeneration. Importantly, this study confirmed that NPY treatment on its own did not exert any adverse effects on retinal cells, demonstrating its nontoxic nature. However, it is crucial to acknowledge that, in some previous studies, NPY, despite its neuroprotective actions, exacerbated damage in a retinal ischemia injury model (22, 55). These effects may be attributed to NPY's role in inducing vasoconstriction through the Y1 receptor, a factor that needs careful consideration in therapeutic applications. The effects on blood flow and perfusion were not investigated in this study. However, given that human glaucoma likely includes a vascular component, understanding NPY's action on the retinal vasculature is critical. This understanding may necessitate designing a receptor-specific peptide to maximize benefits. In conclusion, this study highlighted the effectiveness of NPY treatment in preserving inner retinal health, reducing neuroinflammation, and preventing axonal damage in a glaucoma animal model. This study demonstrated the NPY receptor-mediated activation of the ERK1/2 and PI3K/Akt pathways, which are essential for neuronal survival and proliferation. Further research is essential to improve the delivery of NPY for enhanced therapeutic efficacy. Additionally, it is imperative to investigate the neuroprotective effects of agonists that are specific to NPY receptors in the retina, particularly in the context of glaucoma. This exploration is vital to develop more targeted and effective therapeutic strategies for glaucomatous conditions.

## Materials and methods

### Experimental animals

Wild-type C57BL/6J mice aged 4–6 weeks (body weight 16–21 g) were procured from the Animal Research Centre (ARC) in Perth and Ozgene ARC Pty Ltd in Bentley DC, Western Australia. All animals were housed in an air-conditioned room with maintained temperature (21–28°C), given unrestricted access to food and water, and subjected to 12-h cycles of light and darkness per day. The animals were categorized into four groups (16 mice per group); (i) the control group (normal IOP), (ii) the control group with NPY treatment, (iii) the high IOP (microbead) group, and (iv) the high IOP group with NPY treatment. All animal experiments carried out in this study followed the guidelines specified in the Australian code of practice for the ethical utilization of animals in scientific research and the Association for Research in Vision and Ophthalmology (ARVO) statement for the use of animals in ophthalmic and vision research. The study was approved by the Macquarie University Animal Ethics Committee (AEC reference 2021/026).

### Human retinal lysates

Freshly frozen human cadaver eye samples were obtained from donors with glaucoma and control subjects who consented to the use of their tissues for research purposes (mean age: 74 years; range: 67–82, *n* = 10 per group) from the Sydney Eye Bank in Australia. The Macquarie University Human Research Ethics Committee granted ethical approval.

### Elevated IOP induction via intracameral microbead injections

As established previously, a chronic experimental glaucoma condition was simulated by inducing an elevated IOP in mice by intracameral administration of polystyrene microbeads (10 μm diameter FluoSpheres; Invitrogen, Thermo Fisher Scientific, MA, USA) (56–58). Prior to injection, mice were anesthetized through inhalation of isoflurane (2–5% v/v), in oxygen and maintained on 1–3% isoflurane in oxygen (0.6–1 L/min flow of oxygen), and their pupils were dilated by applying 1% tropicamide (Mydriacyl solution 1%, Alcon Laboratories Pty Ltd, NSW, Australia), followed by the application of topical anesthetic (Alcaine-proxymetacaine 0.5%, Alcon Laboratories Pty Ltd, NSW, Australia). A warming pad was used to maintain the mouse's body temperature during the experimental procedure. The injection was carried out with a 33G needle attached to a 10 µL Hamilton syringe (Hamilton AG), and the procedure was guided by a surgical microscope (Carl Zeiss) to ensure precise alignment. A puncture was made in the cornea, and the needle was inserted at an angle beneath the corneal surface, allowing the gradual release of 2 µL of microbead solution into the anterior chamber. Likewise, the contralateral eye also received a 2 µL saline solution as a control. Post injection, 0.1% dexamethasone (Maxidex, Novartis Pharmaceuticals Australia) and 0.3% ciprofloxacin (Ciloxan; Alcon Laboratories Pty Ltd, NSW, Australia) eye drops were applied to prevent inflammation and infection in the eye after the procedure. Weekly microbead injections were performed to maintain IOP levels above 20 mmHg for 4 weeks. However, in 25% of the animals where IOP declined after the 4th week, further injections were administered in weeks 5 and 6 to sustain this threshold. IOP was measured weekly using a noninvasive rebound tonometer (Icare Tonovet, Helsinki, Finland) while the mice were under anesthesia as described previously (56, 58).

### NPY treatment via intravitreal injection

NPY (ab120208; Abcam, Cambridge, UK) was dissolved in sterile phosphate buffered saline (1× PBS, pH-7.4) at a concentration of 1 mM, stored as aliquots at −80°C, and thawed once before treatments. Following general anesthesia in mice, their eyes were dilated with 1% tropicamide, and a topical anesthetic (Alcaine-proxymetacaine 0.5%, Alcon Laboratories Pty Ltd, NSW, Australia) was subsequently administered. NPY (2 nmols) was carefully injected into the vitreous by inserting the needle at a 45° angle toward the *ora serrata*, posterior to the temporal limbus, while avoiding the lens. The injection was performed using a 33G needle attached to a 10 µL Hamilton syringe (from Hamilton AG), and the procedure was guided by a surgical microscope (Carl Zeiss) to ensure precise alignment. The needle was kept in place for 10 s before removal, allowing the NPY to diffuse and preventing leakage from the injection track. NPY was administered once a week for a duration of 8 weeks.

### Electroretinography

As previously described, pSTR for the control and experimental eyes was recorded by using the Ganzfeld ERG system (Phoenix Research Laboratories, CA, USA) (59–61). Briefly, mice were dark-adapted overnight and later anesthetized with ketamine and medetomidine (75 and 0.5 mg/kg, respectively), before commencing the experiment (*n* = 16 per group). 1% Tropicamide was used to dilate the pupils, and mice were placed on warm pads to maintain the body temperature. The ground electrode was inserted into the tail, and the reference electrode introduced subcutaneously into the animal's forehead. The recording electrode (gold-plate objective lens) was positioned on the eye's corneal surface with lubricant gel to ensure proper contact for ERG recording. A dim stimulus (−4.3 log cd·s/m^2^) was administered 30 times at a 0.5 Hz frequency. The pSTR amplitude was assessed from the baseline to the positive peak at ∼120 ms. Postrecording, atipamezole (1 mg/kg) was injected subcutaneously to reverse the sedation. The mice were then closely monitored following the procedure for their recovery.

### Histological staining

Mice eyes were collected following perfusion with saline and tissue marking dye was used to label the eyeballs, ensuring uniform orientation during the embedding process of the tissues (*n* = 4 per group). Eyes were then fixed in 4% paraformaldehyde (PFA) for 2 hours and washed thrice with 1× PBS. The fixed tissues underwent processing using an automatic tissue processor (ASP200S, Leica, Nussloch, Germany) and later on, embedded in paraffin wax. Fixed whole eye specimens were sectioned into 7 μm thick paraffin slices encompassing the entire retina and ONH in a parasagittal plane. These sections included both the superior and inferior parts of the retina and were then later stained with hematoxylin and eosin (H&E). Images of the stained sections were captured using a Zeiss Axio Imager Z2 microscope and analyzed using ImageJ software (ImageJ, v 1.52; NIH, MD, USA). The cell count in the GCL was conducted across a 500 μm span (ranging from 100 to 600 μm from the optic disc's edge). For each animal eye, cell counts were taken from three sequential sections and then averaged to determine the cell density in the GCL.

### Immunohistochemistry

Mice were euthanized and whole eye tissues, including the optic nerves, were harvested following transcardial perfusion with saline (*n* = 4 per group). The eyeballs were marked with tissue marking dye to ensure consistent orientation during tissue embedding. The eyes were then fixed in 4% PFA for 2 hours and washed three times with PBS. For cryoprotection, the tissues were incubated overnight in 30% sucrose. Subsequently, they were embedded in optimal cutting temperature compound on dry ice and stored at −80°C. A cryostat (Leica) was used to section the whole eye tissues into 10 μm thick sagittal slices and 7 μm thick optic nerve slices (both longitudinal and cross-sections), which were then collected on glass slides. Tissue sections were encircled with a Peroxidase-Antiperoxidase (PAP) pen and incubated for 2 h at room temperature in a blocking buffer containing 5% donkey serum (Sigma-Aldrich, St. Louis, MO, USA) and 0.3% Triton X-100 in PBS. The sections were then incubated overnight at 4°C with the primary antibodies prepared in antibody dilution buffer (3% Bovine serum Albumin (BSA) + 0.3% Triton X-100 in PBS), as listed in Table 1. Post-primary antibody incubation, the sections were washed three times with PBS and incubated with the appropriate secondary antibodies for one hour at room temperature. Following this, the sections were washed three times with PBS and mounted with antifade mounting media containing Prolong DAPI. All images were captured using a Zeiss fluorescence (ZEISS Axio Imager, Carl Zeiss, Oberkochen, Germany). Higher magnification images were captured using a Zeiss LSM 880 fast airyscan confocal microscope. All images were later analyzed using ImageJ software (ImageJ, v 1.52; NIH, MD, USA).

**Table 1.** List of antibodies.

Reagent or resource	Source	Identifier
Primary antibodies		
antiNPY	Cell Signaling technology	Cat#11976; RRID: AB_2716286
antiBrn3a	Millipore Sigma	Cat# MAB1585; RRID: AB_94166
antiNPY1-R	Santa Cruz Biotechnology	Cat# sc-393192; RRID: AB_2721049
antiNPY2-R	Sigma-Aldrich	Cat# SAB4502029; RRID: AB_10747296
antiNPY4-R	Millipore Sigma	Cat# HPA027863; RRID: AB_10602819
antiNPY5-R	Abcam	Cat# ab133757; RRID: AB_2154173
antiGFAP	Abcam	Cat# ab4648; RRID: AB_449329
antiNeuN	Abcam	Cat# ab104225; RRID: AB_10711153
antiIba-1	FUJIFILM	Cat# 019-19741; RRID: AB_839504
antiNeuN	Abcam	Cat# ab104224; RRID: AB_10711040
antipNFH	BioLegend	Cat# 801601; RRID: AB_2564641
antiphospho-Akt (Ser473)	Cell Signaling technology	Cat# XP 4060; RRID: AB_2315049
antiAkt	Cell Signaling technology	Cat# 9272; RRID: AB_329827
antiphospho-p44/42 MAPK (ERK1/2) (Thr202/Tyr204)	Cell Signaling technology	Cat# 4376; RRID: AB_331772
antip44/42 MAPK (ERK1/2)	Cell Signaling technology	Cat# 4695; RRID: AB_390779
antiphospho-mTOR (Ser2448)	Cell Signaling technology	Cat# 2971; RRID: AB_2792875
antiβ-actin	Abcam	Cat# ab6276; RRID: AB_2223210
Secondary antibodies		
Cy3-AffiniPure donkey antirabbit IgG (H + L)	Jackson ImmunoResearch Labs	Cat# 711-165-152; RRID: AB_2307443
Alexa Fluor 488-AffiniPure donkey antimouse IgG (H + L)	Jackson ImmunoResearch Labs	Cat# 715-545-150, RRID: AB_2340846
Horseradish peroxide (HRP) conjugated antirabbit	Jackson ImmunoResearch Labs	Cat# 111–035-003, RRID: AB_2313567
HRP conjugated antimouse	Jackson ImmunoResearch Labs	Cat# 115–035-003, RRID: AB_10015289

### Ex vivo retinal cultures

LY294002 hydrochloride (100 μM) was used to inhibit PI3K activity in retinal explants (62). BIIE 0246 hydrochloride (0.1 µM) was used to block the NPY-Y2 receptor. Different concentrations of CGP 71683 were used to block NPY-Y1 (3 µM), Y4 (6 µM) and Y5 (0.1 µM) receptors. All these reagents were dissolved in DMSO. Mouse retinas were freshly collected in a Dulbecco's modified Eagle's medium containing treatments (*n* = 3 retinas per group). Retinas were separately incubated in PI3K inhibitor and NPY receptor antagonists for 15 min at 37°C. Later, NPY (0.5 µM) was added and further incubated for 45 min at 37°C. After incubation, retinas were washed and lysed for western blotting. Scrambled peptide (scNPY; SKPQRDANREPTRYAIYDYSNPDIELHYLRPAYALG-NH_2_) was synthesized in-house using automated Fluorenyl methoxycarbonyl (Fmoc) solid-phase peptide synthesis on a CEM Liberty Blue peptide synthesizer (CEM, USA) as previously described, and used as a control (63).

### SDS-PAGE and western blotting

Retinal samples were rapidly frozen in liquid nitrogen (*n* = 4 retinas per group) and then resuspended in ice-cold Radioimmunoprecipitation buffer (RIPA lysis buffer) (50 mM Tris, pH 8.0, 150 mM NaCl, 1% Triton X-100, 0.5% sodium deoxycholate) with added 1% phosphatase inhibitor (PhosSTOP) and 1% protease inhibitor cocktail. The tissue suspensions were then sonicated and centrifuged at 12,000 × *g* for 15 min at 4°C to remove debris. The supernatant, containing the total retinal lysate, was collected for protein extraction. Protein concentrations were determined using the Pierce BCA assay kit, with purified BSA (0–2,000 µg/mL) serving as the standard for generating a linear response curve. Protein quantification was performed in triplicate in a 96-well plate, measuring absorbance at 562 nm with a microplate reader, and interpolating unknown protein concentrations from the BSA standard curve. For protein analysis, 20 µg of proteins were subjected to SDS-PAGE on 4–12% gels and transferred to nitrocellulose membranes using electroblotting (Invitrogen iBlot2, Thermo Fisher Scientific, MA, USA) as described previously (56, 58, 64–66). After transfer, membranes were blocked with 5% nonfat dry skim milk powder in Tween-Tris buffered saline (TTBS) (20 mM Tris–HCl pH 7.6, 100 mM NaCl, 0.1% Tween 20) for 1 h at room temperature and incubated overnight at 4°C with primary antibodies, as listed in Table 1. Post-incubation, the membranes were washed thrice with TTBS and incubated with appropriate horseradish peroxidase-conjugated secondary antibodies for an hour at room temperature. Following washes with TTBS, protein bands were visualized using enhanced chemiluminescence (Super Signal West Femto Maximum Sensitive Substrate) as per the manufacturer's instructions. Band images were captured using the ImageQuant LAS 4000 luminescent image analyzer (GE Healthcare, IL, USA), and densitometric analysis of band intensities was conducted using ImageJ software (ImageJ, v 1.52; NIH, MD, USA), with the relative expression of target proteins normalized to β-actin.

### ELISA for NPY quantification in human and mouse retina

NPY levels in retinal lysates were determined using a commercial ELISA kit (Human Neuropeptide Y ELISA, Sigma-Aldrich, St. Louis, MO, USA). The high IOP subjected mice and human postmortem retinal lysates were subjected to ELISA estimation of NPY according to the manufacturer's instructions. A total of 10 human and 8 mouse retinal samples were used for each group in this experiment.

### Quantification and statistical analysis

All data were visualized and subjected to statistical analysis using GraphPad Prism software (version 8.3.0; developed by GraphPad Software Inc., San Diego, CA, USA). Statistical variances among all the groups were assessed using either Student's t test for unpaired data or a one-way ANOVA group analysis. Tukey's multiple comparison post hoc test was used to determine the significance between the groups. All the results are represented as mean ± standard error of the mean (SEM) derived from given *n* sizes. Statistical significance for data analysis was considered with a *P*-value < 0.05.

## Supplementary Material

pgae299_Supplementary_Data

## Data Availability

All data are included in the manuscript.
