# Supplementary material for: Neuropeptide Y receptor activation preserves inner retinal integrity through PI3K/Akt signaling in a glaucoma mouse model
Source: PNAS Nexus. 2024 Jul 26;3(8):pgae299. doi: 10.1093/pnasnexus/pgae299 (PMC11305140; doi:10.1093/pnasnexus/pgae299)
Supplement: pgae299_Supplementary_Data [file pgae299_supplementary_data.pdf]

PNAS nexus

## Supplementary Information for

# Neuropeptide Y Receptor Activation Preserves Inner Retinal Integrity Through PI3K/Akt Signaling in a Glaucoma Mouse Model

Viswanthram Palanivel[1*], Vivek Gupta[1*], Nitin Chitranshi[1], Ole Tietz[2], Roshana Vander Wall[1], Reuben Blades[2], Kanishka Pushpitha Maha Thananthirige[2], Akanksha Salkar[1], Chao Shen[3], Mehdi Mirzaei[1], Veer Gupta[4], Stuart L Graham[1,5], Devaraj Basavarajappa[1].

[1]Macquarie Medical School, Faculty of Medicine, Health and Human Sciences, Macquarie University, North Ryde, Sydney, NSW 2109, Australia.

[2]Dementia Research Centre, Macquarie Medical School, Faculty of Medicine, Health and Human Sciences, Macquarie University, North Ryde, Sydney, NSW 2109, Australia.

[3]Microscopy Unit, Faculty of Science and Engineering, Macquarie University, North Ryde, Sydney, NSW 2109, Australia.

[4]School of Medicine, Deakin University, Geelong, VIC 3216, Australia.

[5]Save Sight Institute, The University of Sydney, Sydney, NSW 2000, Australia.

* Viswanthram Palanivel and Vivek Gupta

**Email:** viswanthram.palanivel1@hdr.mq.edu.au and vivek.gupta@mq.edu.au

**This PDF file includes:**

Supplementary text (Materials)
Figure S1

**Supplementary Information Text**

**Materials**

NPY and ScNPY were diluted in carbonate-bicarbonate coating buffer (50mM, pH 9.6) to a final concentration of 10 µg/ml and coated onto Nunc microtiter plates (Thermo Fisher Scientific, USA) for overnight incubation at 4°C. The coated plates were then washed with PBS containing 0.05% Tween 20 (Sigma Aldrich, MO, USA) and blocked with 5% bovine serum albumin (BSA) for 2 hours at room temperature (RT). Mouse retina lysates (n=4), diluted to 10 µg/ml, were added to the NPY-coated plates and incubated for 2 hours at RT. After washing with wash buffer, specific primary antibodies (anti-NPY-Y1R, Y2R, Y4R, and Y5R) were added to separate wells and incubated for 1 hour at 37°C. Following another wash, HRP-linked secondary antibodies were incubated for 30 minutes at 37°C. The reactions were quenched and detected using 3,3′,5,5′-Tetramethylbenzidine (TMB) substrate, and the absorbance was read at 450 nm using a microplate reader (CLARIOstar Plus, BMG Labtech, Ortenberg, Germany). Data were analyzed using GraphPad Prism (version 8.3.0; developed by GraphPad Software Inc., San Diego, CA, USA).

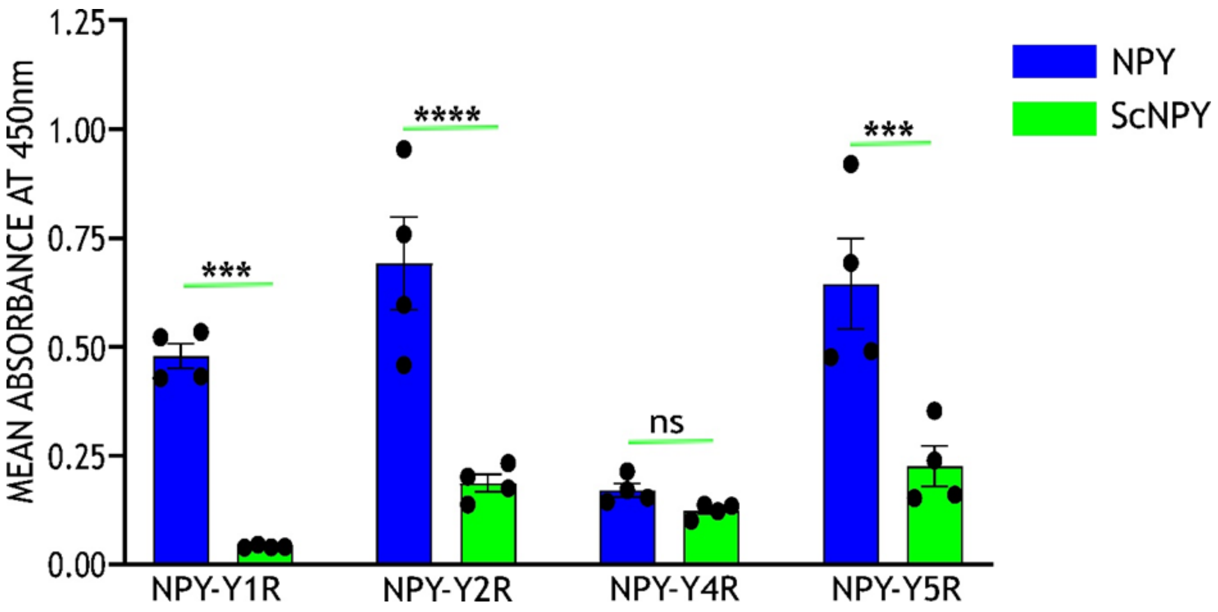


**Fig. S1. Competitive ELISA to determine the NPY and its receptor binding interactions.**

A significant decrease in absorbance was observed with ScNPY (green) compared to NPY (blue) in the NPY-Y1R (***p<0.001), NPY-Y2R (****p<0.0001), and NPY-Y5R (***p<0.001) groups, while no significant difference was found between NPY and ScNPY in the NPY-Y4R group. Statistical significance was determined using one-way ANOVA (F(7,24) = 19.14, n=4) with Tukey’s multiple comparisons test. Results are presented as mean ± SEM.
